# Cavernous Sinus Dural Arteriovenous Fistula Patients Presenting With Headache as an Initial Symptom

**DOI:** 10.14740/jocmr2489w

**Published:** 2016-02-27

**Authors:** Motohiro Nomura, Kentaro Mori, Akira Tamase, Tomoya Kamide, Syunsuke Seki, Yu Iida, Yuichi Kawabata, Tatsu Nakano, Hiroshi Shima, Hiroki Taguchi

**Affiliations:** a Department of Neurosurgery, Yokohama Sakae Kyosai Hospital, Yokohama, Japan; b Department of Neurology, Yokohama Sakae Kyosai Hospital, Yokohama, Japan; c Department of Neurosurgery, Shima Neurosurgical and Orthopedic Clinic, Kawasaki, Japan; d Department of Neurosurgery, Taguchi Neurosurgery Clinic, Yokohama, Japan

**Keywords:** Headache, Migraine, Dural arteriovenous fistula, Cavernous sinus

## Abstract

Cavernous sinus (CS) dural arteriovenous fistula (dAVF) patients presenting with only headache as an initial symptom are not common. Patients with CS-dAVF commonly present with symptoms related to their eyes. In all three patients, headache was the initial symptom. Other symptoms related to the eyes developed 1 - 7 months after headache. In one patient, headache was controlled by sumatriptan succinate, but not diclofenac sodium or loxoprofen sodium. In another patient, headache was controlled by loxoprofen sodium. In the third patient, headache was improved by stellate ganglion block. In all patients, magnetic resonance angiography (MRA) in the early stage of the clinical course showed abnormal blood flow in the CS. However, reflux to the superior ophthalmic vein (SOV) was not detected. As treatment, transarterial and transvenous embolizations were necessary for one patient, and transvenous embolization was performed for another patient with significant blood flow to the SOV and cortical veins. On the other hand, manual compression of the bilateral carotid arteries at the neck resulted in disappearance of the fistula in the third patient. In all patients, the symptoms improved after the disappearance of blood reflux to the CS. The refluxed blood to the CS might cause elevation of the CS pressure and stimulate the trigeminal nerve in the dural membrane, resulting in headache before developing reflux in an anterior direction. CS-dAVF could induce both migraine and common headache. In cases with blood reflux to the CS on magnetic resonance imaging and/or MRA even without eye symptoms, a differential diagnosis of CS-dAVF should be taken into consideration.

## Introduction

A dural arteriovenous fistula (dAVF) is a lesion with an arteriovenous shunt in the dural membrane. Patients with dAVF in the cavernous sinus (CS) usually present with symptoms related to the eyes, such as exophthalmos, chemosis, and palpebral swelling [1-3]. For CS-dAVF patients presenting with only headache or facial pain as an initial symptom, the diagnosis of CS-dAVF is not easy. In this report, we present three patients with CS-dAVF whose initial symptom before developing visual symptoms was headache, focusing on its clinical course and radiological findings.

## Case Reports

### Case 1

**Figure 1:**
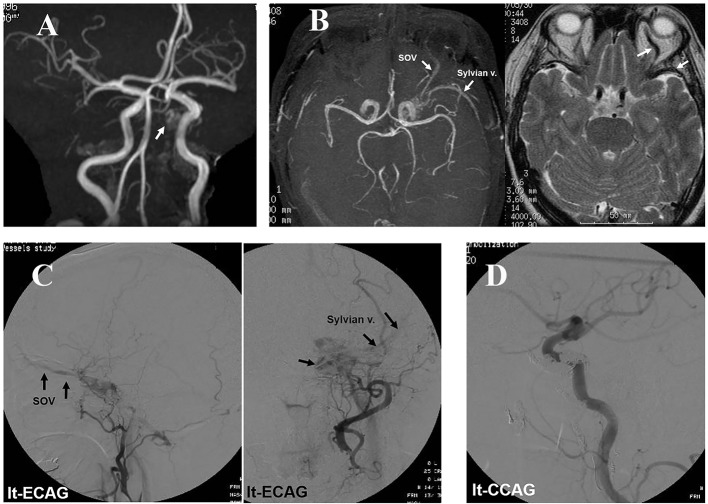
Case 1: (A) MRA obtained 3 months after the initial headache showing an abnormal intensity in the left CS (arrow), but no dilatation of the SOV. (B) MRI demonstrating blood reflux to the left CS, SOV, and Sylvian vein after the patient had become aware of eye symptoms at the eighth month. (C) Angiography showing CS-dAVF reflux to the left SOV and Sylvian vein. (D) Post-embolization angiography showing no blood reflux to the CS.

A 61-year-old woman transiently experienced mild headache. About a month later, she experienced left-sided headache and pain around the left orbit, and visited our hospital. Computed tomography (CT) revealed no abnormality. Her headache was controlled by sumatriptan succinate, but not diclofenac sodium or loxoprofen sodium. Magnetic resonance angiography (MRA) obtained 3 months after the initial headache showed an abnormal intensity in the left CS (Fig. 1A). However, magnetic resonance imaging (MRI) and MRA did not show blood reflux or dilatation of the superior ophthalmic vein (SOV) or a cortical vein. Her headache improved 3 months after the onset and she remained asymptomatic for a few months. About 7 months after the initial headache, she became aware of chemosis, palpebral swelling, and pain around the orbit on the left side. She consequently visited our hospital again. MRI obtained 8 months after the initial headache showed blood reflux to and dilatation of the left SOV and Sylvian vein (Fig. 1B). Angiography (Fig. 1C) demonstrated that the right CS was fed by the abnormal blood flow from the right internal carotid artery (ICA). The refluxed blood to the right CS flowed into the left CS via the intercavernous sinus. The left CS was opacified by the blood flow via the left ICA and branches of the left external carotid artery (ECA). Blood in the left CS refluxed to the left SOV and Sylvian vein. As a treatment, transarterial embolization (TAE) with platinum coils of the left maxillary artery was initially performed. Eight days later, the left middle meningeal and ascending pharyngeal arteries were also embolized with platinum coils. However, the lesion did not disappear on angiography, and the symptoms remained. Therefore, transvenous embolization (TVE) of the shunt in the left CS was conducted via the left inferior petrous sinus (Fig. 1D). After TVE, her visual symptoms and headache disappeared. She has not experienced further headache, and MRI has revealed no recurrence of the lesion for 6 years.

### Case 2

**Figure 2:**
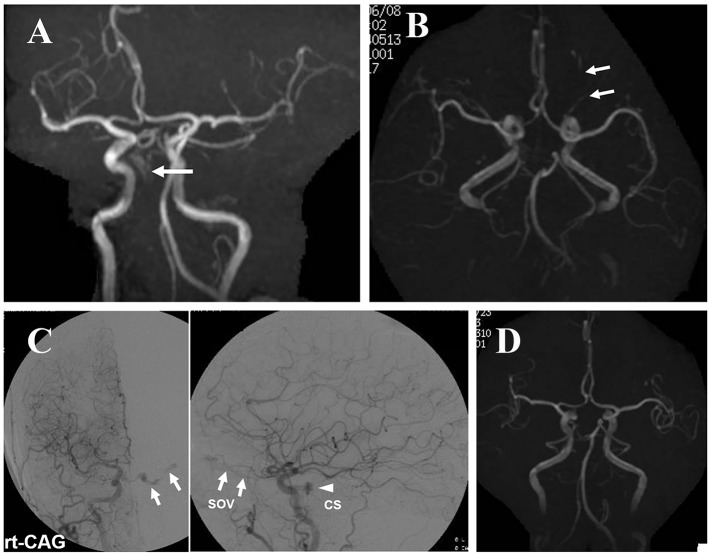
Case 2: (A) MRA performed 1 month after the initial headache demonstrating an abnormal intensity in the right CS (arrow). Reflux to and dilatation of the SOV are not apparent. (B) MRI after the patient had become aware of double vision, again showing blood reflux to and dilatation of the left SOV (arrows). (C) Angiography showing arteriovenous shunt to the right CS and reflux to the left SOV. No blood reflux to a cortical vein is observed. (D) MRA after manual compression of the carotid arteries showing the disappearance of blood reflux to the left SOV.

A 68-year-old woman experienced headache in the right temporal region. She visited our hospital 15 days after the initial symptom. Her headache was controlled by loxoprofen sodium and gradually disappeared. MRA obtained 1 month after headache showed abnormal blood flow in the right CS. However, blood reflux to a cortical vein or dilatation of the SOV was not detected (Fig. 2A). She experienced double vision 1.5 months after the initial headache. The double vision spontaneously improved 2 months later. For several months, she had no headache or double vision. However, at the eighth month, she became aware of double vision again, and visited our hospital. MRI showed blood reflux to and dilatation of the left SOV, but not a cortical vein (Fig. 2B). Angiography (Fig. 2C) demonstrated blood reflux to the right CS from the right ICA. The refluxed blood to the right CS flowed into the left CS via the intercavernous sinus and further refluxed to the left SOV. The left CS was also opacified with blood flow from the left carotid artery. The blood reflux was not severe, and reflux to a cortical vein was not observed. Therefore, manual compression of the bilateral carotid arteries at the neck was performed by the patient. After 3 months, MRI revealed that CS-dAVF had disappeared (Fig. 2D). No recurrence of symptoms or the lesion has been detected for 8 years.

### Case 3

**Figure 3:**
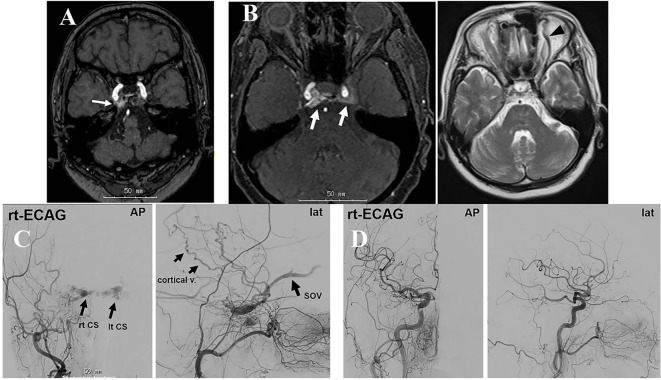
Case 3: (A) MRI obtained 2 months after the initial pain demonstrating an abnormal intensity in the right CS (arrow). (B) MRI/MRA obtained 10 months after onset showing abnormal blood flow in the bilateral CS (arrows) and dilatation of the left SOV (arrowhead). (C) Angiography showing abnormal blood flow in the right CS and SOV. The refluxed blood flows to the left CS via the intercavernous sinus and further to the left SOV. (D) Post-embolization angiography showing the disappearance of the abnormal blood flow.

A 74-year-old woman experienced headache in the right temporal region. One month later, she experienced right facial pain and double vision. MRI obtained 2 months after the initial headache in another hospital showed an abnormal intensity in the right cavernous sinus (Fig. 3A). Three months later, the double vision disappeared; however, the right facial pain remained. At the fifth month, she had severe headache and visited another local hospital. Repeated MRI also showed an abnormal intensity in the right cavernous sinus. However, the abnormality was not recognized by the physician. She was referred to a pain clinic and stellate ganglion block (SGB) was conducted. Headache gradually improved and disappeared with SGB. After that, she did not experience headache for about 3 months. At the ninth month, she became aware of right palpebral swelling, chemosis, and lacrimation. Ten months after the initial symptom, she visited a neurosurgical clinic, and right exophthalmos was additionally pointed out. MRI revealed abnormal blood flow in the right CS (Fig. 3B). She was referred to our department. Angiography (Fig. 3C) demonstrated abnormal blood flow to the right CS from the right ICA and branches of the right ECA. The refluxed blood flowed to the contralateral CS via the intercavernous sinus, and further to the bilateral SOV and right cortical veins. The condition was diagnosed as right CS-dAVF. TVE using platinum coils was performed 12 months after the initial headache. The posterior part of the right CS where the abnormal blood shunts were present was embolized and the abnormal blood flow disappeared (Fig. 3D). After TVE, her symptoms related to the eyes disappeared.

## Discussion

CS-dAVF has some draining routes including both anterior and posterior directions. CS-dAVF shows symptoms according to its draining direction. Here, we report three patients with CS-dAVF whose initial symptom was only headache. In patients with only headache or facial pain, a diagnosis of idiopathic inflammation of the CS (Tolosa-Hunt syndrome) may be made [4], and diagnosis of dAVF is not easy. In all patients, MRI obtained in early stage of the clinical course showed abnormal blood flow in the CS. However, reflux to the SOV was not detected, and the patients had no visual symptoms. The refluxed blood to the CS might cause CS pressure elevation, stimulate the trigeminal nerve in the dural membrane, and induce headache [5]. After a while, the blood refluxed anteriorly to the SOV, and visual symptoms including double vision and disturbance of eyeball movement become apparent. In our three patients, symptoms related to the eyes became apparent 1 - 7 months after the initial headache. The initial MRA showed an abnormal intensity in the CS suggesting arteriovenous shunt. However, dilatation of the SOV was not seen at this time. In such a situation in the early stage without eye symptoms, a diagnosis of CS-dAVF is not easy. The abnormal intensity in the CS might not be paid sufficient attention. Follow-up MRI clearly showed blood reflux to the SOV in all patients, and to the Sylvian vein in two patients. Blood reflux to the cortical veins may cause intracerebral hemorrhage. Therefore, an early diagnosis of CS-dAVF is necessary. In patients with headache and an abnormal intensity in the CS on MRI or MRA even without eye symptoms, a differential diagnosis of CS-dAVF should be considered.

In one patient, headache was only controlled by sumatriptan succinate, indicating that the headache was a migraine. Recently, Osaka et al [6] reported a patient with CS-dAVF whose headache was improved by sumatriptan. According to them, the mechanism is due to a triptan-induced reduction in shunting flow via vasoconstriction. In another patient, it was improved by a non-steroidal anti-inflammatory drug, suggesting a common headache. In the third patient, headache was improved by SGB. There is a report describing the effect of SGB on migraine [7]. The mechanism of SGB to control migraine is suggested to be the stabilization of sympathetic nerves, or suppression of edema and inflammation of the vascular wall [8]. Our observations indicated that CS-dAVF could induce both migraines and common headaches prior to the appearance of eye symptoms.

As for radical treatment, TVE with or without TAE was necessary for two patients with significant blood reflux to the cortical veins. TVE is an effective treatment to cure the condition. In one patient, manual compression of the bilateral carotid arteries at the neck improved the shunts and symptoms. In all three patients, the headache improved after the disappearance of the blood shunt.

### Conclusions

CS-dAVF could induce both types of headache, a migraine and common headache. Close observation focusing on the existence of dAVF is necessary in patients with abnormal CS intensities on MRI or MRA, even though the symptom is only headache.
